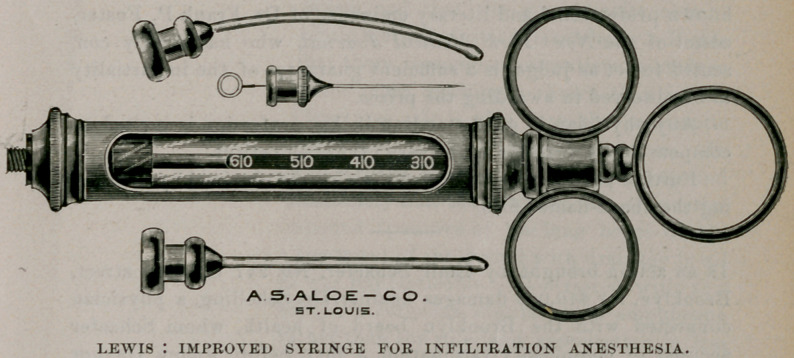# Miscellany

**Published:** 1896-01

**Authors:** 


### Miscellany.

DR. C. L. SCHLEICH, of Berlin, has experimented with reference to topical anesthesia obtained by infiltration. Dr. Weller Van Hook, of Chicago, has published clinical notes on the subject. Messrs. John Wyeth & Brother, manufacturing chemists, Philadelphia, have made tablets adapted to this method that tend to insure the accuracy of its application. The formulas used are those suggested by the authors above named, and the tablets can be obtained through druggists or of the manufacturers direct.

Dr. Bransford Lewis, of St. Louis, has devised an improved syringe for infiltration anesthesia, according to the method of Schleich. Dr. Lewis, in describing the instrument, an illustration of which we publish, says : That, no matter how easily or satisfactorily it may be employed in the superficial structures, where there are no large vessels in danger of being punctured with the hypodermic needle, when one is injecting in the depths of a

LEWIS : IMPROVED SYRINGE FOR INFILTRATION ANESTHESIA.

wound in the neighborhood of large arteries or veins, as in enucleating bubo-glands immediately above the femoral vessels, the likelihood of running the needle into one of them and producing disastrous results is not a fancied one.

By means of the needles represented in the cut, he has been enabled to do away with this difficulty. They are blunt-pointed, and made of German silver, so that, though of sufficient stiffness to be thrust into the connective tissues of a wound after the skin has been severed, they would not injure a blood-vessel if pushed against one.

The anesthesia is begun, therefore, with the sharp steel needle, and continued with either of the two silver ones. The choice between the latter depends on whether a curved or straight needle is more conveniently used. This instrument is made by A. S. Aloe Co., of St. Louis, Mo. Armed with the tablets of Wyeth and the syringe of Lewis, the physician is prepared to practise the method of Schleich with safety and accuracy.

---

Six Hundred ($600) Dollars in Prizes.—The special attention of our readers is called to the advertisement of the Palisade Manufacturing Co., Yonkers, N. Y., with the above title, on second page of cover of this issue.

The prize contest which this well-known firm announces will no doubt attract attention, and result in the submission of many papers of merit on the clinical value of antiseptics, both internal and external. The prizes are extremely liberal, and the well-known professional and literary eminence of Dr. Frank P. Foster, editor of the *New York Medical Journal*, who has kindly consented to act as judge, is a sufficient guarantee of the impartiality to be observed in awarding the prizes.

Any physician in good standing in his profession is invited to compete on equal terms with every other competitor.

Further particulars as to conditions can be obtained by addressing the above-named firm.

---

In an action brought by Emil Schaefer, No. 241 Suydam street, Brooklyn, for $10,000 damages against Dr. Schelling, a physician connected with the Brooklyn board of health, whom Schaefer accused of compulsory vaccination, a jury before Judge Gaynor recently brought in a verdict of $1,500 for the plaintiff.

---

The Samuel D. Gross Prize.—The second quinquennial prize of $1,000 under the will of the late Samuel D. Gross, M. D., will be awarded January 1, 1900. The conditions annexed by the testator are that the prize "shall be awarded every five years to the writer of the best original essay, not exceeding 150 printed pages, octavo in length, illustrative of some subject in surgical pathology or surgical practice, founded upon original investigations, the candidates for the prize to be American citizens.

The essays, which must be written by a single author in the English language, should be sent to Dr. J. Ewing Mears, 1429 Walnut street, Philadelphia, before January 1, 1900.

## Figures and Tables

**Figure f1:**